# Anxious parents show higher physiological synchrony with their infants

**DOI:** 10.1017/S0033291720005085

**Published:** 2022-10

**Authors:** C. G. Smith, E. J. H. Jones, T. Charman, K. Clackson, F. U. Mirza, S. V. Wass

**Affiliations:** 1 Institute of Psychiatry, Psychology and Neuroscience, King's College London, London, UK; 2 Birkbeck, University of London, London, UK; 3 University of Cambridge, Cambridge, UK; 4 University of Plymouth, Plymouth, UK; 5 University of East London, London, UK

**Keywords:** Anxiety, infant, parent, perinatal mental health, physiology, stress regulation, synchrony

## Abstract

**Background:**

Interpersonal processes influence our physiological states and associated affect. Physiological arousal dysregulation, a core feature of anxiety disorders, has been identified in children of parents with elevated anxiety. However, little is understood about how parent–infant interpersonal regulatory processes differ when the dyad includes a more anxious parent.

**Methods:**

We investigated moment-to-moment fluctuations in arousal within parent-infant dyads using miniaturised microphones and autonomic monitors. We continually recorded arousal and vocalisations in infants and parents in naturalistic home settings across day-long data segments.

**Results:**

Our results indicated that physiological synchrony across the day was stronger in dyads including more rather than less anxious mothers. Across the whole recording epoch, less anxious mothers showed responsivity that was limited to ‘peak’ moments in their child's arousal. In contrast, more anxious mothers showed greater reactivity to small-scale fluctuations. Less anxious mothers also showed behaviours akin to ‘stress buffering’ – downregulating their arousal when the overall arousal level of the dyad was high. These behaviours were absent in more anxious mothers.

**Conclusion:**

Our findings have implications for understanding the differential processes of physiological co-regulation in partnerships where a partner is anxious, and for the use of this understanding in informing intervention strategies for dyads needing support for elevated levels of anxiety.

## Introduction

Research has shown continuity of lifetime anxiety disorders from parents to children: multiple anxiety disorders pose a significant risk of anxiety in offspring (Lawrence, Murayama, & Creswell, 2019). However, while anxiety disorders aggregate in families, the reasons for this are still not yet understood (Murray, Creswell, & Cooper, 2009). Genes associated with an underlying liability towards current anxiety symptoms across the population are largely shared with those predisposing individuals to professionally-diagnosed lifetime anxiety disorder (Purves et al., 2020), yet evidence acknowledges the key role of environmental influences in the development of anxiety (Eley et al., 2015). Early childhood has been found to be a crucial period for identifying environmental risk factors for anxiety disorder (Möller, Nikolić, Majdandžić, & Bögels, 2016), including the potential for early identification of high-risk individuals, and for preventative, early interventions. The present study examines, therefore, how anxious symptoms in parents relate to affect co-regulation in parent-infant dyads.

In both anxious and non-anxious families, there is considerable evidence that parents play a positive role in regulating children's physiological, behavioural and affective states (Bridgett, Burt, Edwards, & Deater-Deckard, 2015; Reddy, Hay, Murray, & Trevarthen, 1997). Behavioural studies have, for example, identified sensitive parenting behaviours that mediate the relationship between household chaos and infant self-regulatory skills (Vernon-Feagans, Willoughby, & Garrett-Peters, 2016), and parental encouragement mediates the relationship between parent anxiety and anxiety symptoms in early childhood (Murray et al., 2008, 2009). Physiological studies examining how autonomic arousal co-fluctuates in infant-parent dyads have traditionally concentrated on physiological synchrony, referring to a range of temporally interdependent or associated activities in the physiological processes of two partners (Davis, West, Bilms, Morelen, & Suveg, 2018; McFarland, Fortin, & Polka, 2020). Previous research has suggested that the benefits of synchrony are bidirectional (Feldman, 2007): the parent, by adapting to the child, helps by responding contingently to the child's needs (Feldman, 2009); the child, by adapting to the parent, gains both self-control, and self-awareness (Feldman, Greenbaum, & Yirmiya, 1999). Previous research has identified synchronous patterns of change in physiological arousal in the lab following the administration of experimental stressors (Ham & Tronick, 2009). Recent research that recorded naturalistic arousal co-fluctuations in infant-parent dyads found that synchronous patterns of co-fluctuating arousal were not observed across all arousal states: rather, that short-term increases in parent-child synchrony were triggered in response to ‘peak’ instances of physiological arousal in the infant, but that synchrony at other times was not observed (Wass et al., 2019a, 2019b).

There is also substantive evidence that anxious parenting can associate with the dysregulation of behavioural and physiological states in children (Nikolić et al., 2016). Behavioural studies examining tabletop play between anxious parents and their infants found evidence for an ‘overloaded, highly stimulating’ behavioural profile in anxious mothers (Feldman, 2007), along with higher levels of behavioural synchrony (Beebe et al., 2011; Granat, Gadassi, Gilboa-Schechtman, & Feldman, 2017). Anxiety in these studies was measured via self-report questionnaire. Experimental investigations have also shown overactive regulatory responses from infants of anxious mothers, particularly following the onset of positive social stimuli (Granat et al., 2017). Lab-based physiological studies have found evidence for ‘stress contagion’, whereby increases in autonomic activity in the mother are reflected in increases in the infant following emotionally-valenced experimental tasks (Waters, West, & Mendes, 2014, 2017). However, naturalistic investigations of physiological synchrony between infants and parents with anxiety are minimal.

Overall, studies of maternal anxiety and physiological dysregulation in early childhood remain scant. Arousal dysregulation (often defined as increased autonomic changes in response to an experimentally administered challenge, along with longer recovery times; e.g., Beauchaine and Thayer, 2015) is a core feature of anxiety in adulthood (Ottaviani et al., 2016; Thayer, Friedman, & Borkovec, 1996) and middle childhood (Dieleman et al., 2015; Koszycki, Taljaard, Bialejew, Gow, & Bradwejn, 2019), but the majority of research on this topic focuses on children aged 6 or over (Siess, Blechert, & Schmitz, 2014). In addition, these findings examine change relative to a stressor, with a discrete and experimenter-defined start and end period, administered during short periods (~<10 min) of lab-based interaction. No previous research has examined whether spontaneous fluctuations in a child and parent's biological and behavioural systems associate with one another in naturalistic, day-to-day settings, assessing how these relationships differ between more or less anxious parents. Additionally, while emotion dysregulation is also characteristic of anxiety disorders (Amstadter, 2008; Hofmann, Sawyer, Fang, & Asnaani, 2012), there has been little study into the relationship between affect states and physiological dysregulation in mother–infant pairs where the mother has anxiety. One issue with measuring hyperarousal alone is that its valence cannot be determined; to resolve this, vocal signals of positive or negative affect may be used to identify valence (as in previous work showing that extremes of valence are more likely at elevated levels of arousal; see Wass et al., 2019a, 2019b). To our knowledge, no previous research has disaggregated infant recovery from an instance of physiological hyperarousal with positive or negative valence and examined whether the relationship between infant recovery and maternal reactivity to positive or negative hyperarousal events varies by maternal anxiety.

To address this, we developed new techniques, including miniaturised microphones, video cameras, electro-cardiograms, and actigraphs that could be worn concurrently by infants and parents for a day at a time at home (Maitha et al., 2020; Wass et al., 2019a, 2019b). We recorded both partners' autonomic fluctuations during the day, by measuring heart rate (RR intervals, where R is the peak of the QRS complex of the ECG wave), heart rate variability, and movement (via actigraphy). According to previous research, elevated heart rate, decreased heart rate variability and increased movement are associated with increased physiological stress, i.e. a higher ratio of sympathetic to parasympathetic nervous system activity (Cacioppo, Tassinary, & Bernston, 2007). We also recorded the auditory environment and coded the vocalisations spoken by the infant, and those directed to the infant by the parent.

The goal of the current study was to examine associations between the physiological profiles for infant-parent dyads with higher or lower measures of maternal anxiety. In the analyses of two partners' time series data, a well-established distinction has been drawn between ‘concurrent’ synchrony (‘when A is high, B is high’) and ‘sequential’ synchrony (‘changes in A forward-predict changes in B’ – see Wass, Whitehorn, Marriott Haresign, Phillips, & Leong, 2020). Given previous evidence, we asked a set of four interlinked questions from around 4 hours per dyad of continuously measured parent and child arousal data:

First (hypothesis 1), we examined the degree of concurrent and sequential infant-parent arousal synchrony across the full time series of home-based data from each dyadic pair. We predicted that both forms of synchrony would be greater in dyads with more anxious parents.

Second, across the next three analyses, we examined how overall levels of dyadic synchrony relate to structured variation in the degree of synchrony across the time series. In hypothesis (2), we asked how sequential synchrony varies in relation to the current levels of arousal in both dyadic partners, considered at the same time – and we examined how this differs by parental anxiety level. We predicted that arousal changes in each partner considered independently would be influenced by the overall level of arousal of the dyad and that this relationship would differ contingent on parental anxiety.

Third, since previous research (Wass et al., 2019a, 2019b) has shown that synchronous responses may be constrained to highly stressful events, we went on to focus the analysis on moments when the infant showed a peak in their arousal (hypothesis 3). We predicted that more anxious parents would show greater event-related physiological hyper-arousal.

Finally, peak arousal events in the infant could be positive or negative in affect. In hypothesis (4) we predicted that parents' event-related hyperarousal would associate with infants' hyperarousal across different emotionally valenced events.

## Method

### Experimental participant details

The project was approved by the Research Ethics Committee at the University of East London. Participants were recruited from the London, Essex, Hertfordshire and Cambridge regions of the UK. In total, 91 infant-parent dyads were recruited to participate in the study, of whom usable autonomic data were recorded from 82. Of these, usable paired autonomic data (from both parent and child) were obtained from 74 participants. Of these, 68 of these participants also completed the full anxiety screening questionnaire. A consistent outlier-detection strategy was applied equally for all analyses, by excluding outliers that were >2 inter-quartile range (IQR) from the mean, to avoid violating the assumptions of the statistical tests being conducted. Outliers were only found for the analyses presented under hypotheses 1 and 2, reported below in the Results. Further details, including exclusion criteria, and detailed demographic details on the sample, are given in Table 1 and SM section 1.1. Of note, we excluded families in which the primary day-time care was performed by the male parent because the numbers were insufficient to provide an adequately gender-matched sample. All participating parents were, therefore, female. Participants received £30 in Love2Shop gift vouchers as a token of gratitude for participation, split over two visits.

**Table 1.** Demographic data split by low/high parental GAD-7 scores

	Low anxiety	High anxiety
Infant age (days) – mean (s.d.)	349 (39)	370 (41)
Gender (*N* (%) male)	14 (42)	13 (39)
Infant ethnicity *N* (%)		
White British	17 (51)	16 (47)
Other white	1 (4)	1 (4)
Afro-Caribbean	4 (11)	3 (9)
Asian, Indian and Pakistani	2 (7)	5 (16)
Mixed – White/Afro-Caribbean	1 (4)	1 (4)
Mixed – White/Asian	0 (0)	4 (11)
Other mixed	3 (9)	4 (11)
Household income (%)		
Under £16k	9 (27)	10 (31)
£16–£25k	8 (24)	10 (31)
£26–£35k	7 (20)	3 (9)
£36–£50k	4 (11)	4 (11)
£51–£80k	4 (11)	2 (7)
>£80k	1 (4)	3 (9)
Maternal education (%)		
Postgraduate	12 (36)	9 (27)
Undergraduate	19 (56)	16 (47)
FE qualification	0	1 (4)
A-level	0	2 (7)
GCSE	3 (9)	1 (4)
No formal qualifications	0	2 (7)
Other	0	2 (7)

### Parent screening

To screen parents for maternal anxiety, participants filled out the Generalized Anxiety Disorder 7-item screener (GAD-7), which assesses anxiety symptoms over the past 2 weeks (Spitzer, Kroenke, Williams, & Löwe, 2006). Responses were given on a 4-point scale ranging from 0 (not at all) to 3 (nearly every day). Validity for this questionnaire has been provided by studies with clinical and non-clinical populations, with scores above 6 representing moderate anxiety (Löwe et al., 2008). The internal consistency of the scale was *α* = 0.89.

The mean (s.d.) (range) of scores obtained on the GAD-7 was 3.4 *(3.9)* (0–17). A median split was performed to differentiate between high and low anxiety groups. The dichotomization of this variable was necessary due to our statistical analysis plan (in particular, our use of time series analyses), though additional analyses based on a quintile split were used to explore the consistency of associations (see SM 2.1). The mean (s.d.) (range) GAD-7 score was 0.76 *(0.85)* (0–2) for the low anxiety group and 6.16 *(3.96)* (3–17) for the high anxiety group, indicating mild to moderate anxiety.

### Experimental method details

Participating parents were invited to select a day during which they would be spending the entire day with their child but which was otherwise, as far as possible, typical for them and their child. The researcher visited the participants' homes in the morning (c. 7.30–10 am) to fit the equipment, and returned later (c. 4–7 pm) to pick it up. The mean (s.d.) recording time per day was 7.3 *(1.4)* hours.

**Fig. 1.**
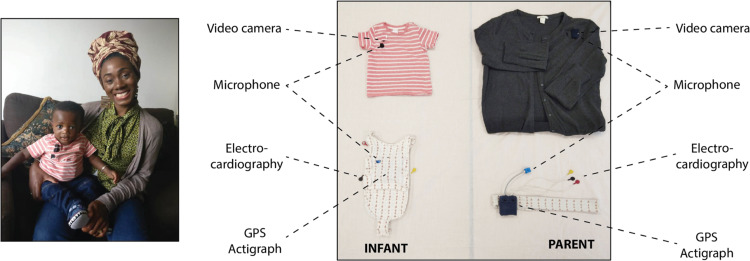
Left – illustration of parent and child wearing the equipment; right – the equipment used for home monitoring.

The equipment consisted of two wearable layers, for both infant and parent (see Fig. 1). For the infant, a specially designed baby-grow was worn next to the skin, which contained a built-in electrocardiogram (ECG) recording device (recording at 250 Hz), accelerometer (30 Hz), Global Positioning System (1 Hz), and microphone (11.6 kHz). A T-shirt, worn on top of the device, contained a pocket to hold the microphone and a miniature video camera (a commercially available Narrative Clip 2 camera). For the parent, a specially designed chest strap was also worn next to the skin, containing the same equipment. A cardigan, worn as a top layer, contained the microphone and video camera. The clothes were comfortable when worn and, other than a request to keep the equipment dry, participants were encouraged to behave exactly as they would do on a normal day. To ensure good quality recordings, the ECG device was attached using standard Ag-Cl electrodes, placed in a modified lead II position.

### Quantification and statistical analysis

#### Autonomic data parsing and calculation of the autonomic composite measure

Further details on the parsing of the heart rate, heart rate variability, and actigraphy are given in the SM (section 1.2). To ensure the accuracy of these recording devices, they were cross-validated by recording heart rate and heart rate variability using both the new devices at home and established recording devices (a Biopac MP150 amp recording at 2000 Hz) in lab settings. High reliability was observed both for heart rate (*ρ* = 0.57, *p* < 0.001) and heart rate variability (*ρ* = 0.70, *p* = 0.01). In the SM (section 1.3), we also present further details on our motivation for collapsing these three measures into a single composite measure of autonomic arousal.

#### Affect coding

The microphone recorded a 5-s snapshot of the auditory environment every 60 s. *Post hoc*, coders identified samples in which the infant was vocalising, and coded them for vocal affect on a scale from 1 (fussy and difficult) to 9 (happy and engaged). In order to assess inter-rater reliability, 24% of the sample was double coded; Cohen's kappa was 0.60, which is considered acceptable (McHugh, 2012). All coders were blind to intended analyses. Negative affect vocalisations were defined as all vocalisations coded as 4 or less; positive affect vocalisations included all vocalisations coded on 6 or more; neutral affect vocalisations include vocalisations coded 5.

#### Home/awake coding

Our analyses only examine segments of the data in which the dyad was at home, and the infant was awake. This is because our preliminary analyses suggested that infants tended to be strapped in to either a buggy or car seat for much of the time that they were outdoors, which strongly influenced their autonomic data. Further details for how these home/awake segments were identified are given in SM section 1.4. Following these exclusions, the mean (s.d.) total amount of data available per dyad was 3.7 *(1.7)* hours, corresponding to 221.5 *(102.4)* 60-second epochs per dyad.

#### Cross-correlation analyses

To test hypothesis 1, we used cross-correlation to examine relations between concurrently measured epochs of parent and infant arousal. Infant and adult arousal data were synchronized, 60-s epoched and linear de-trended. Spearman's rank order correlations were conducted across all pairs of time-locked (i.e. simultaneously occurring) epochs for infant and parent and plotted as time ‘0’ (*t* = 0). Correlations between non-simultaneous pairs were then computed and plotted against time lag and direction on the *x*-axis (adult's arousal forward-predicting infant arousal on the positive axis, infant arousal forward-predicting adult arousal on the negative). Figures present data for a selected epoch of 600 s before to 600 s after an event to fully contextualize profiles of change around the focal point (see Thorson, West, and Mendes, 2018). Permutation-based temporal clustering analyses were applied to correct for multiple comparisons across time bins (see below, and SM section 1.6 for more details).

#### Vector plots

To test hypothesis 2, we computed vector plots. To do this, all infant and adult arousal data were downsampled into 60-s epochs and collated into six equally sized bins, individually for each participant (infant and adult). Each epoch was then classified according to what bin it fell into for both infant arousal and parent arousal. This is represented as a two-dimensional matrix – so all epochs that were bin 3 for infant arousal and bin 4 for adult arousal are drawn at location (*x* −3, *y* −4). The size of each dot within the matrix indicates what proportion of the total available samples was located within each bin. For each bin, we then calculated the average change from all epochs in that bin, to the epochs immediately following. This change score is drawn on the vector plot as a red line. Thus, for the point located at (6, 6) on the vector plot, which represents all epochs that were classified as in bin 6 for both infant arousal and parent arousal, the vector extends −0.8 on the *x*-axis (representing a change in infant arousal), and −1 on the *y*-axis (representing a change in adult arousal). This indicates that across all epochs starting from (6, 6), the average change to the next epoch was a reduction of 0.8 bins in infant arousal, and 1 bin in adult arousal.

These plots, therefore, allow us to examine how the parent's present arousal level *interacts with* the child's present arousal level in predicting the change in parent arousal – i.e. how the change in one partner's arousal is influenced by both partners' arousal, considered in combination. To quantify this, we compared the change in adult arousal between the bottom right (high infant-low adult arousal) and bottom left (low infant-low adult arousal) quadrants of the Vector plot; and between the top right (high infant-high adult arousal) and top left (low infant-high adult arousal) quadrants of the Vector plot. The observed results are compared to a chance value of 0 using a *t* test.

#### Permutation-based temporal clustering analyses

To estimate the significance of time series relationships, a permutation-based temporal clustering approach was used for the analyses presented under hypotheses 1, 3, and 4. This procedure, which is adapted from neuroimaging (Maris, 2012; Maris & Oostenveld, 2007), allows us to estimate the probability of temporally contiguous relationships being observed in our results, a fact that standard approaches to correcting for multiple comparisons fail to account for (Maris & Oostenveld, 2007; see also Oakes, Baumgartner, Barrett, Messenger, & Luck, 2013). For further details, see SM section 1.5.

## Results

### Raw data and descriptives

**Fig. 2.**
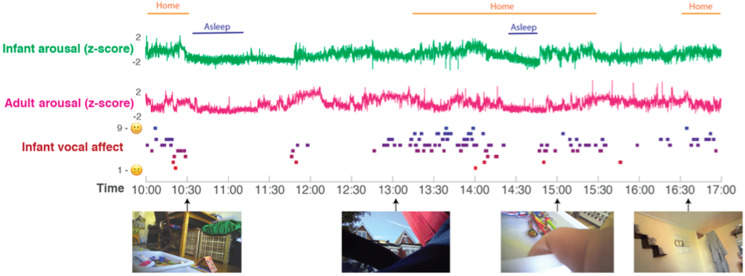
Raw data sample. A sample day's data from a single dyad is shown. Time (from 10 am to 5 pm) is shown on the *x*-axis. From top to bottom: the home/awake coding; the infant and parent arousal composites (see SM section 1.1); infant vocal affect; sample frames from the data recorded from the camera. All measures are calculated as described in the Methods section.

Prior to testing our four main hypotheses, we first present raw data and descriptive analyses. Figure 2 shows a raw data sample of the home data, and Table 1 shows demographic data for the sample, subdivided by low/high GAD-7 scores. Independent samples *t* tests were conducted for all demographic variables (i.e. with the exception of ethnicity) to assess whether significant group differences were observed. No significant differences were identified (all *p*s > 0.15).

As a preliminary analysis, we examined how the low/high GAD-7 groups differed on mean arousal levels across the day. This analysis was based on the raw autonomic data included in the arousal composite, prior to the calculation of *z* scores on a per-participant basis for the composite measure. When considering just samples in which the dyad was at home, and the infant was awake, *t* tests indicated no differences between the lower/higher anxiety groups on any of the heart rate variables included in the *z*-scored composite, namely mean waking heart rate, sleeping heart rate, waking or sleeping heart rate variability, for either infants or parents (all *p*s > 0.27). Hence, arousal levels did not differ significantly between the groups. Waking movement levels were, however, significantly lower in the high GAD-7 group *t*(69) = 2.17, *p* = 0.03.

*Hypothesis 1*: concurrent and sequential infant-parent synchrony in physiological arousal is greater in dyads with more anxious parents

To test this hypothesis, we examined the cross-correlation between infant and parental arousal. Prior to conducting the *t* test group comparisons described below, two outliers (one from each group) were excluded using the >2 inter-quartile range (IQR) criterion.

**Fig. 3.**
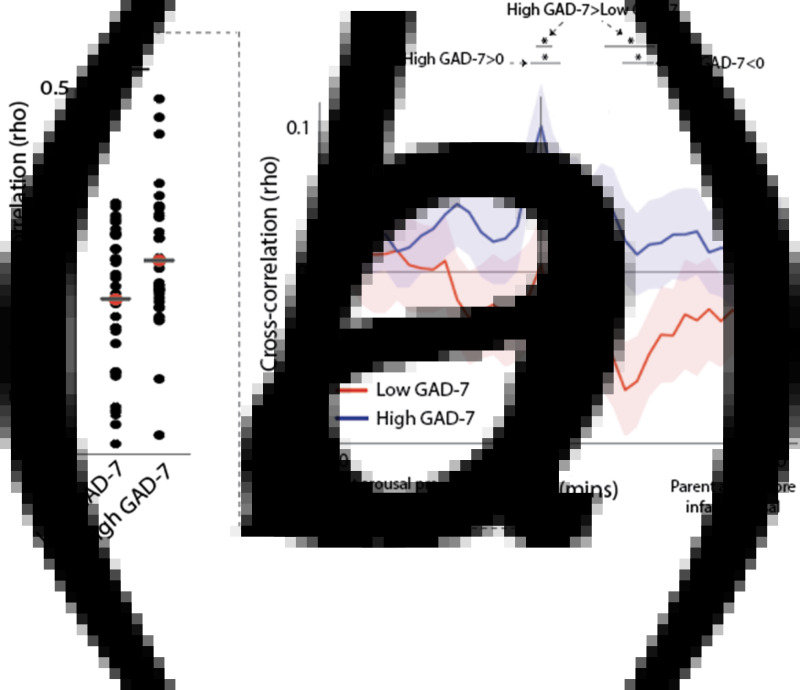
(*a*) Scatterplot showing the zero-lagged cross-correlation between parent and child arousal, subdivided by maternal anxiety (i.e. low and high GAD-7). * indicates the results of the *t* tests conducted as described in the main text *p* < 0.05. (*b*) cross-correlation function between parent and child arousal, subdivided by low and high parental anxiety. The peak at time 0 indicates that when parent and infant arousal synchrony are compared, they significantly associate and this is greater in high anxiety dyads than low anxiety dyads. Shaded areas indicate the standard error of the means. * *p* < 0.05 following correction for multiple comparisons using permutation-based temporal clustering analyses (see SM section 1.5).

In previous research, we used an identical analysis to show that, across all parents, no significant temporal co-fluctuation in infant and parental autonomic arousal levels is observed (Wass et al., 2019a, 2019b). When results are subdivided by parental anxiety, however, a significant zero-lagged cross-correlation between infant and parent arousal is observed in the anxious group [*t* test *v.* chance value of 0 (*t*(32) = 4.2, *p* < 0.001)] but not the non-anxious group [*t*(32) = 1.03, *p* = 0.32 (Fig. 3*a*)]. Group comparisons indicated higher zero-lagged cross-correlations in Group 1 *v.* Group 2: *t*(64) = 2.16, *p* = 0.035. In sum, when considering all home-awake segments of the day, there is significant co-fluctuation in autonomic arousal between parent and child arousal in the high GAD-7 but not the low GAD-7 group.

Further details and interpretation of the cross-correlation function are given in SM section 2.1. In this section, we also provide a further analysis subdividing our groups using a quintile split by GAD-7 score (see online Supplementary Fig. S2). This shows the relationship between arousal cross-correlation and GAD-7 is distributed uniformly across the sample and highest in participants with the most elevated levels of anxiety.

*Hypothesis 2*: arousal changes in each partner will be influenced by the overall arousal level of the dyad; this relationship will differ contingent on parental anxiety

Hypothesis 1 examined differences in arousal synchrony across all data collected while the dyad was at home and the infant was awake. In addition, we also wished to examine how inter-dyadic influences in arousal would vary contingent on the arousal level of parent and child, considered separately. To examine this, we calculated a vector plot (see the section Methods).

**Fig. 4.**
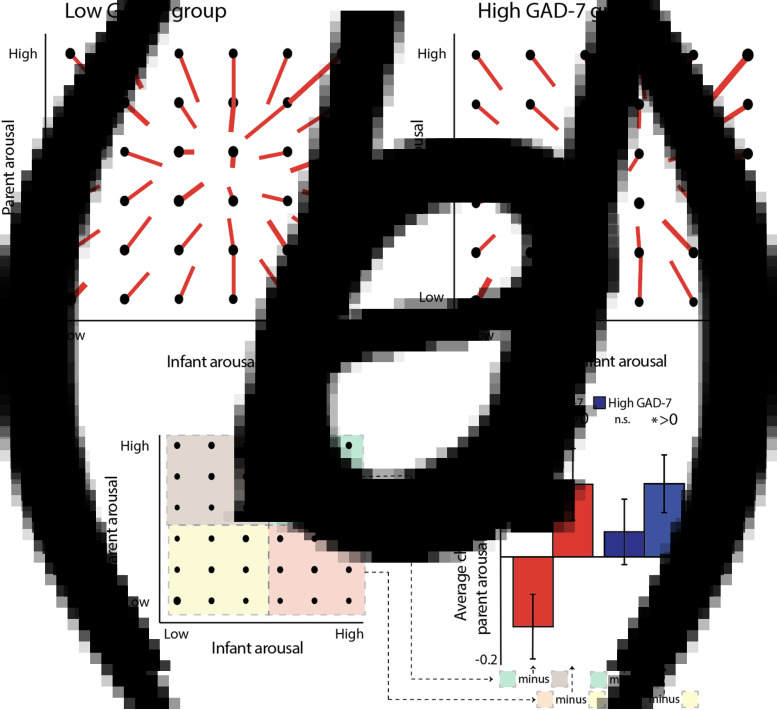
*(a)*–(*b*) Vector plot illustrating transitions between arousal bins, contingent on starting arousal state. (*a*) Shows non-anxious (low GAD-7) group; *(b)* shows anxious (high GAD-7) group. Data were averaged into 60-s epochs and binned from 1 (low) to 6 (high), for infant and parent separately. Thus, an epoch classified as (1, 1) indicates an epoch in which both infant and parent were in a low arousal state. The red line indicates the average direction of travel between that and the subsequent epoch, averaged across all epochs in that bin. Thus, for the position (1, 1) on plot *(a)*, the red line shows a displacement of +0.3 on the *x*-axis and +0.4 on the *y*-axis, indicating that the average epoch starting at (1, 1) showed an increase of +0.3 in infant arousal and +0.7 in adult arousal to the subsequent epoch. *(c)* schematic illustrating the analysis whose results are shown in (d). Each vector plot was divided into four quadrants: Parent low/Infant low (yellow, *1*), Parent low/Infant high (red, *2*), Parent high/Infant low (brown, *3*), and Parent high/Infant high (green, *4*). In order to investigate how infant arousal and adult arousal interacted to predict the change in adult arousal, we subtracted the average adult change scores in quadrant *4* from quadrant *3*, and quadrant *2* minus quadrant *1*. This was performed separately for the two groups. *(d)* bar chart showing the results of the analysis: when the adult's arousal starts high, their arousal decreases more in instances where the infant's arousal is high, than when it is low (low GAD-7 group only). * indicates the significance of the analyses comparing the observed values to a chance level of 0. *p < 0.05, †*p* = 0.05.

The parent-infant arousal change score is drawn on the vector plot as a red line. For example, for the point located at (1, 1) on the vector plot (Fig. 4*a*), the vector extends +0.3 on the *x*-axis (representing a change in infant arousal), and +0.7 on the *y*-axis (representing a change in adult arousal). Hence, across all epochs starting from (1, 1), the average change to the next epoch was a gain of +0.3 bins in infant arousal, and +0.7 bins in adult arousal (see Fig. 4*a*, *b*). Across all data, the vectors tend to point towards the centre of the plot. This indicates regression to the mean: in an epoch where infants' and parents' arousal starts low, an increase is expected to the next epoch; whereas for an epoch that starts high, a decrease is expected. The centre point of the vectors appears to be around bin 4 (out of 6), consistent with the lightly positively skewed distribution observed across all data (see Wass et al., 2019a, 2019b).

In order to examine how the change in one partner's arousal is influenced by both partners' arousal considered in combination, we can examine, for example, the bottom rows of each vector plot (Fig. 4*a*, *b*), which show instances in which the adult's arousal is low. The bottom left quadrant (shaded yellow on Fig. 4*c*) shows instances in which both parent and infant arousal is low; the bottom right quadrant (shaded red) shows instances in which the parent arousal is low but infant arousal is high. To estimate whether, in both groups, the change (increase) in adult arousal is greater where the infant arousal is high than when it is low, we calculated the change in adult arousal between the bottom right and bottom left quadrants of the vector plot (Fig. 4*c*), and compared the observed results to a chance value of 0 using a *t* test. Results from four participants were excluded (three low/one high) using the ±2IQR rule. For both the low [*t*(30) = 2.03, *p* = 0.05] and high [*t*(32) = 2.39, *p* = 0.02] GAD-7 groups, marked differences from zero were observed. Hence, when adults' arousal is low and infants' arousal is high, then adults show upregulation in their arousal in response – a feature which is present in both the low and high GAD-7 groups.

The top rows of the vector plot (Fig. 4*c*, *d*) show instances in which the adult's arousal is high. In the non-anxious group, it appears that the negative vertical displacement of the lines is greater in the top right quadrant (shaded green on Fig. 4*c*), compared to the top left quadrant (shaded brown). If true, this would indicate that, when the adult's arousal starts high, their arousal *decreases* more in instances where the infant's arousal is high than when it is low. To estimate this, we calculated the change between quadrants and compared the observed results to a chance value of 0 using a *t* test. Results from three participants were excluded (one low/two high) using the ±2IQR rule. For the lower anxiety [*t*(32) = 2.16, *p* = 0.04] but not the higher anxiety [*t*(31 = 0.75, *p* = 0.46] groups, a significant difference was observed. An independent samples *t* test also identified a significant difference between groups on this measure [*t*(63) = 2.05, *p* = 0.045]. Hence, when the overall arousal level of the dyad is high, then adults show downregulation in their arousal in response – but this feature is only present in the low GAD-7 group.

*Hypothesis 3*: more anxious parents will show greater event-related physiological hyperarousal

**Fig. 5.**
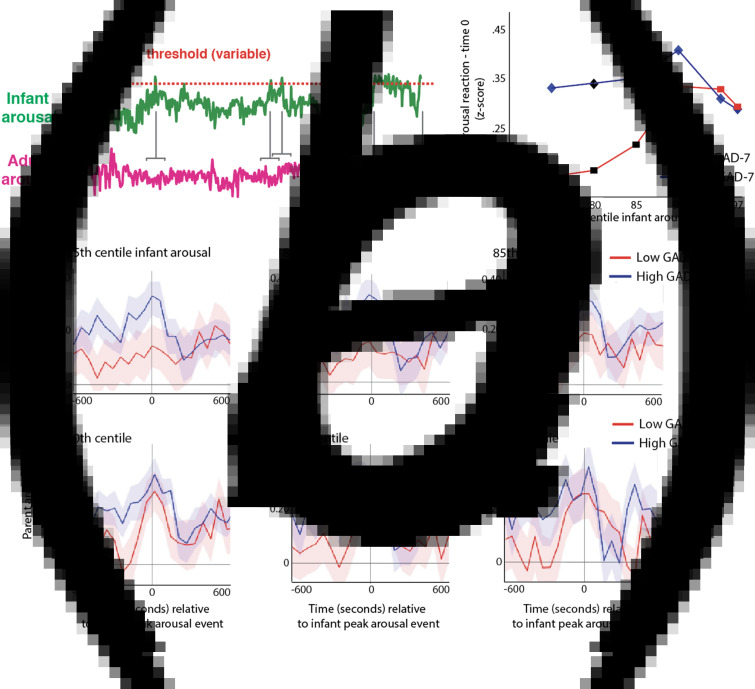
*(a)* Schematic illustrating the analysis shown in *(b)*–*(c)*. First, adult's arousal data are *z*-scored, participant by participant. Next, instances where the infant's arousal crosses a centile threshold (e.g. exceeded the 95^th^ centile of samples for that infant in that day) were identified. Then, for each instance, the change in adult arousal from 600 s before to 600 s after the infant peak arousal moment was excerpted. Individual instances were averaged to index how the adult's arousal level changed relative to the ‘peak’ arousal moment of the infant. The analysis was repeated using different values for the centile threshold. *(b)* Change in parent arousal relative to ‘peak’ arousal moments of the infant, defined using variable centile thresholds. *(c)* Summary plot showing just the time 0 parent arousal levels from the plots in (b). Both groups showed maternal reactivity to extremes of infant arousal, but high GAD-7 parents showed greater autonomic reactivity to small-scale fluctuations in infant arousal. Where the permutation-based temporal clustering analyses indicated that a significant peak in adult arousal was observed relative to the infant ‘peak’ arousal event, the datapoint has been drawn in colour (blue/red for anxious/non-anxious group, i.e. high/low GAD-7 groups). Where no significant peak in adult arousal was observed, the datapoint has been drawn black. It can be seen that the lower anxiety group only show significant peaks in parent arousal relative to the 3% and 5% most extreme instances of elevated infant arousal; but the higher anxiety group show significant peaks in parent arousal relative to the 25%, 15%, 10%, 5%, and 3% most extreme instances.

Hypothesis 1 examines parent-infant synchrony, i.e. the continuous association between parent and infant arousal across all data. In addition and motivated by previous findings (Wass et al., 2019a, 2019b), we also examined adult reactivity to ‘peak’ arousal events from the infant. Figure 5*a* shows a schematic illustrating this analysis. First, adult's arousal data were *z*-scored, participant by participant. Next, instances, where the infant's arousal crossed a centile threshold (e.g. exceeded the 97^th^ centile of samples for that infant in that day) were identified. Then, for each instance, the average change in adult arousal from 600 s before to 600 s after the infant peak arousal moment was excerpted (see Fig. 5*b*). This allows us to examine how the adult's arousal changes on average around the top 3% most elevated arousal moments for that infant on that day. Then, we repeated the analysis using different values for the centile threshold (Fig. 5*b*), to examine instances where the infant's arousal exceeded the 95^th^ centile of samples for that infant on that day, the 90^th^ centile, and so on, down to the 75^th^ centile.

We were interested to examine whether a significant peak in parent arousal was observed relative to the peak arousal moment in the infant, and whether peaks in parent arousal were only observed for the most extreme instances of elevated infant arousal (i.e. the top 3% of samples for that infant in that day), or whether they were also observed for less extreme, yet still relatively high, arousal instances (i.e. the top 25% of the sample for that infant that day). To quantify whether a significant peak in parent arousal was observed relative to the peak arousal moment in the infant, we performed a permutation-based clustering analysis (see SM section 1.6, Method 1). Instances where a significant peak was observed are drawn as coloured datapoints on Fig. 5*c* (blue/red for high/low GAD-7 groups); instances where no significant peak was observed are drawn as black datapoints. It can be seen that, after correction for multiple comparisons, the low GAD-7 group only show peaks in parent arousal relative to the 3% and 5% most extreme instances of elevated infant arousal. In contrast, the high GAD-7 group show significant peaks in parent arousal relative to the 25%, 15%, 10%, 5%, and 3% most elevated instances. Overall, these results show that both groups showed maternal reactivity to extremes of infant arousal, but that high GAD-7 parents also showed greater autonomic reactivity to less extreme arousal fluctuations in the infant.

*Hypothesis 4*: parents' event-related hyperarousal associates with infants' hyperarousal across different emotionally valenced events

Hypothesis 3 examines how adults react to naturally occurring ‘peak’ moments in infant arousal during the day. However, high arousal levels can be positive or negative, and differently valenced infant arousal may make a difference to parent responsivity. To examine this, we also studied hyperarousal relative to vocalisations, which signal whether infants are experiencing positive or negative emotional valence. We examined how parents' event-related hyperarousal associates with infants' hyperarousal across different emotionally valenced events.

**Fig. 6.**
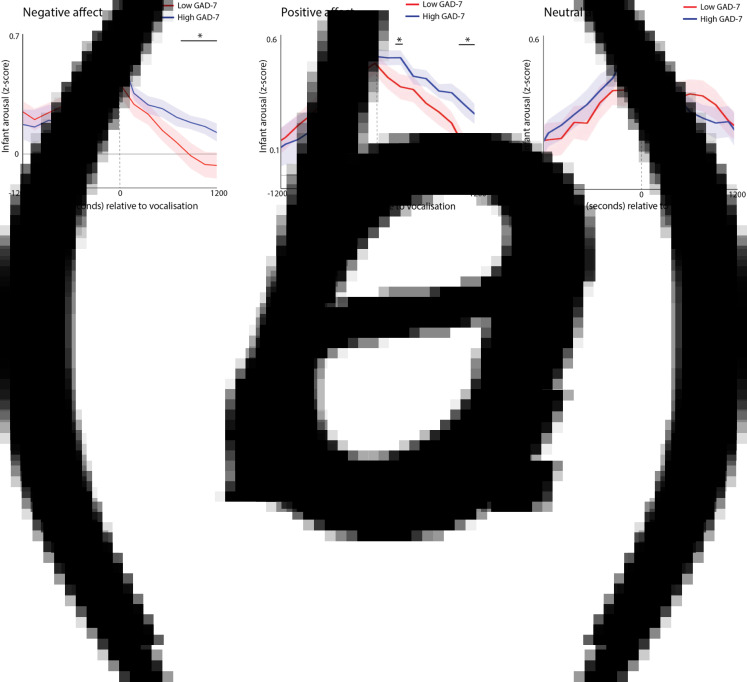
Change in infant autonomic arousal relative to *(a)* negative affect vocalizations; *(b)* positive affect vocalizations; *(c)* neutral affect vocalizations. For each plot, the blue line shows the anxious group (High GAD-7), and the red line the non-anxious group (Low GAD-7). The high GAD-7 group show significantly higher infant physiological arousal at the time of the negative and positive (but not neutral) vocalisation at time 0, along with significantly high arousal 8–12 min afterwards. Shaded area shows standard errors. Areas identified as showing above-chance group differences following correction for multiple comparisons using the permutation-based clustering analysis are highlighted with *.

First, we identified all infant vocalisations that occurred during the day; for each vocalisation, we examined the rate of change of infant physiological arousal relative to these vocalisations (Fig. 6*a*, *c*). The significance of group differences was calculated by first conducting *t* tests separately for each individual time bin, and then correcting for multiple comparisons using a permutation-based clustering analysis (see SM section 1.6, Method 2). As expected, all vocalisations showed a significant peak in infant autonomic arousal at time 0 – i.e. the time of the infant vocalisation (all permutation-based clustering *p*s < 0.001). The infants with high anxiety mothers showed significantly higher infant physiological arousal at the time of the negative affect vocalisation, along with significantly higher infant arousal during the period 8–12 min after the vocalisation, indicating slower recovery (Fig. 6*a*, *p* = 0.023). A similar pattern was evident following positive affect vocalisations (Fig. 6*b*, *p* < 0.001), but not following neutral affect vocalisations. These differences were not attributable to differences in the frequency of vocalisations as these did not differ significantly between groups (*z* = 0.31/1.50/.97, *p* = 0.75/0.30/0.33 for negative/positive/neutral affect vocalisations, respectively).

We also wished to assess how infant recovery following a positive or negative vocalisation related to the differences in parental reactivity to moments of peak infant arousal examined in hypothesis 3, above. To do this, we measured the degree to which maternal autonomic reactivity is specific to ‘peak’ infant arousal moments, using the following method. For each participant, the maternal arousal response to >97^th^ centile infant arousal moments was calculated (see Fig. 5*b*). This was done by averaging the *z*-scored maternal arousal values from 3 min before and after the peak infant arousal moment (corresponding to the peaks visible on Fig. 5*b*; as seen in Fig. 5, analyses were also repeated using other time windows with similar results). For each participant, the maternal arousal response to >75^th^ centile arousal moments was also calculated (see Fig. 5*b*). The degree to which maternal autonomic reactivity is specific to ‘peak’ infant arousal moments was calculated by subtracting the >97^th^ centile arousal responses from the >75^th^ centile responses so that a larger value indicates that maternal autonomic reactivity is more specific to ‘peak’ infant arousal moments.

Infant recovery was assessed by calculating the average infant arousal during the period from 1200 s before and after the positive and negative affect vocalisations (corresponding to the time periods shown in Fig. 6), and subtracting the average arousal during the period after the vocalisation from the average arousal during the period before. In order to assess how infant recovery related to parental reactivity, we calculated the bivariate correlation between the two measures. Infant recovery following negative affect related to more selective parental reactivity (i.e. a bigger difference between >97^th^ centile and >75^th^ centile arousal responses): ϱ = −0.33 *p* = 0.045. This finding was observed consistently in the lower (ϱ = −0.31) and higher (ϱ = −0.50) parental anxiety groups. No relationship was observed between the same variable and infant recovery following positive affect (ϱ = −0.07). These results show that more selective parental autonomic reactivity is associated with faster infant recovery following naturally occurring peaks of negative affect – a finding which is observed independently in both the low and high GAD-7 groups.

## Discussion

The present study aimed to examine how anxious symptoms in parents relate to arousal co-regulation in parent-infant dyads. Primarily, we investigated whether concurrent and sequential synchrony in physiological arousal would be greater in dyads with more anxious parents (Hypothesis 1). We also examined how inter-dyadic influences in arousal vary contingent on the starting arousal level of parent and child, considered separately (Hypothesis 2). In addition, we examined patterns of event-related change (sequential synchrony). We examined whether more anxious parents show greater event-related changes in their own physiological arousal, relative to ‘peak’ moment of arousal in the child (Hypothesis 3). And we examined whether parents' event-related hyperarousal associates with infants' hyperarousal across different emotionally valenced events (Hypothesis 4). To address these questions, we used miniaturised microphones and cameras, and wearable physiological monitors, to record vocalisations and day-long physiological fluctuations in 12-month-old infants and their parents. Participating parents completed a self-rating scale of current anxiety symptoms (the GAD-7).

Our preliminary analyses indicated that mean heart rate and heart rate variability did not differ between the more or less anxious groups for either parent or infants in home settings. This is informative, because no previous research has, to our knowledge, examined baseline (resting) physiology in an infant proband sample. We did however, find differences in how arousal levels in dyads associated with each other throughout the day. Overall, dyads in the more anxious group showed higher concurrent synchrony in physiological arousal (Hypothesis 1). Conversely, in the less anxious group, mothers' arousal levels were less tightly coupled with infant levels (Fig. 3*b* and SM section 2.1).

Recent research has reported correlated neural activity between socially interacting animals (Kingsbury et al., 2019; Zhang & Yartsev, 2019) consistent with previous neuroimaging findings in adults (Hari, Himberg, Nummenmaa, Hämäläinen, & Parkkonen, 2013; Hasson, Ghazanfar, Galantucci, Garrod, & Keysers, 2012). Our results extend this by identifying, for the first time, *higher* physiological synchrony in anxious parent-child dyads. Although our finding is consistent with some previous evidence on behavioural synchrony in anxious dyads (Beebe et al., 2011; Granat et al., 2017), the finding of greater physiological synchrony is novel. This finding contributes to a growing evidence base suggesting that ‘sustained intervals of synchrony may be too demanding from a resource allocation perspective’ (McFarland et al., 2020, p. 58), and that a mid-range of synchrony whereby partners are neither over- nor under-coordinated is optimal (Beebe et al., 2011; Granat et al., 2017; Jaffe et al., 2001). This is important for understanding mechanisms for direct transfer of physiological stress across parent-child dyads.

Also novel is our finding examining how parents react to small- *v.* large-scale arousal fluctuations in their child (Hypothesis 3). Our results showed that, for the non-anxious group, significant peaks in adult arousal were observed only relative to the top 5% and top 3% most elevated instances of infant arousal, whereas, anxious parents show peaks in arousal also relative to the top 25%, 15%, and 10% most elevated instances of infant arousal (key to this finding is that anxious parents exhibited a significant *change in arousal* – rather than greater arousal overall). This suggests that, whereas non-anxious parents up-regulate their own arousal only relative to ‘peak’ arousal moments in their infant, more anxious parents show greater reactivity to small-scale fluctuations in their child. Thus, non-anxious mothers were ‘there when you need me’ – showing reactivity to peak child arousal events, but not otherwise. But anxious mothers were ‘always on’ – showing reactivity to small-scale child arousal fluctuations as well. In Hypothesis 4, we found that more selective parental reactivity is associated with faster infant recovery following naturally occurring peaks of negative affect – a finding which is observed independently in both the low and high anxiety groups. These findings support evidence for an ‘overloaded, highly stimulating’ behavioural profile in anxious mothers (Feldman, 2007), that leaves insufficient time for infants to experience neutral affect, or ‘time off’, thereby losing opportunities to practice self-regulation.

Finally, our results provide new evidence on how anxious parents' arousal levels change depending on their own and their infant's starting arousal level (Hypothesis 2). Our results suggested that, when adults' arousal is low and infants' arousal is high, then adults tend to upregulate their arousal in response – a feature which is present in both the low and high anxiety groups. But, when the overall arousal level of the dyad is high, then adults tend to downregulate their arousal in response – a feature which is only present in the lower anxiety group. This latter feature potentially indicates behaviours akin to ‘stress buffering’ (Hennessy, Kaiser, & Sachser, 2009); this behaviour was absent among more anxious mothers. Our findings suggest that the mechanism by which affective and arousal states are transmitted from one partner to another does not operate consistently across more anxious and less anxious dyads, and may therefore be a fruitful target for further research.

Our research is limited by several factors. Firstly, our sample was sourced from the community. Subgroup analyses (see SM section 2.1, online Supplementary Fig. S2) suggested that the relationship between arousal cross-correlation and GAD-7 was distributed uniformly across the sample, and highest in participants with most severe anxiety, although the elevated levels of anxiety found in clinical samples were relatively under-represented in our sample. Of note, there is genetic evidence that total GAD-7 scores have the same genetic underpinnings as professionally diagnosed anxiety disorders (Purves et al., 2020). Though trait scores of anxiety may be more pertinent to the general population than clinical diagnosis and have broader relevance in terms of effects, further research with a clinical sample would be needed to investigate the effects of moderately severe and severe levels of anxiety in mothers.

A second limitation of our study is that we investigated biobehavioural relations between mother-infant dyads and not father-infant dyads; research has suggested that gender differences in parents are relevant for childhood anxiety disorders and should be a focus in the future (Majdandžić, Möller, de Vente, Bögels, & van den Boom, 2014; Möller, Majdandžić, & Bögels, 2015). A third limitation is that, though we requested participants select a typical day for the home recording session, we had no way of confirming the typicality of the day chosen; as such, there was no way to know if state anxiety, as well as trait anxiety, could be exerting an effect on parent or infant arousal. Finally, our research did not differentiate anxiety subtypes, for example general anxiety disorder *v.* panic disorder or social anxiety disorder; evidence suggests children respond differentially to parents on these bases, and therefore these subtypes should be incorporated into future research among mother-infant dyads (de Rosnay, Cooper, Tsigaras, & Murray, 2006; Murray, Cooper, Creswell, Schofield, & Sack, 2007).

Our research provides new information on how the regulatory profiles of anxious mother-infant dyads are inter-dependent on one another. It also contributes to the evidence-base on the intergenerational transmission of anxiety from parent to infant, building on our understanding of how parent–child interactions differ in anxious parents during the first year of life. The research also provides evidence that even in mothers without a professional diagnosis of anxiety, there are apparent effects of maternal anxiety on physiological processes in both mother and infant. This information is helpful for developing our knowledge of the environmental mechanisms underlying the development of anxiety disorders, and provides a basis for future investigations into *how* an individual partner might downregulate another's arousal levels. It may also inform future intervention studies focused on reducing overall levels of anxiety in the dyad, whether or not the parent has a clinical diagnosis; for example, targeting interoceptive capacities in the parent.

## Supplementary material

For supplementary material accompanying this paper visit https://doi.org/10.1017/S0033291720005085. click here to view supplementary material
