# Supplementary material for: Anxious parents show higher physiological synchrony with their infants
Source: Psychol Med. 2021 Feb 10;52(14):3040–50. doi: 10.1017/S0033291720005085 (PMC9693696; doi:10.1017/S0033291720005085)
Supplement: Supplementary file 1 [file S0033291720005085sup001.docx]

##

Supplementary Materials for:

**Anxious parents show higher physiological synchrony with their infants**

Table of Contents

[1 Supplementary Methods 1](#_Toc52359649)

[1.1 Experimental participant details 1](#_Toc52359650)

[1.2 Additional information for parent screening 1](#_Toc52359651)

[1.3 Autonomic data parsing 1](#_Toc52359652)

[1.4 Justification for use of composite autonomic arousal measure 2](#_Toc52359653)

[1.5 Home/Awake coding 3](#_Toc52359654)

[1.6 Calculation of permutation-based temporal clustering analyses 3](#_Toc52359655)

[2 Supplementary Results 5](#_Toc52359656)

[2.1 Hypothesis 1 – Synchrony between infant and parent arousal – differences contingent on parental anxiety – further analyses 5](#_Toc52359657)

# 1 Supplementary Methods

## Experimental participant details

This sample size was selected prior to the commencement of the study based on power calculations presented, and approved by peer review, in the funding application that supported this work (ESRC ES/N017560/1). Exclusion criteria included: complex medical conditions, skin allergies, heart conditions, parents below 18 years of age, and parents receiving care from a mental health organisation or professional.

## Additional information for parent screening

The relationship between increased exposure to stressful life events and risk of general anxiety disorder is well established (Blazer et al., 1987; Francis et al., 2012; Muhsen et al., 2008). A life events scale was therefore administered, calculating the quantity of stressors (e.g. serious illness or injury, bereavement, abuse, unemployment, relocation, contact with the criminal justice system) experienced by the participant or immediate family in the previous 12 months. In line with expectations, participants in the high anxiety group indicated significantly higher quantities of stressful life events, *t*(76) = -3.30, *p* = .001.

## Autonomic data parsing

Electro-Cardiography data were parsed to identify RR intervals using custom-built Matlab scripts, employing an adaption of a standard thresholding procedure (Wass et al., 2015), and verified *post hoc* via visual inspection*.* Heart Rate Variability was calculated using the PhysioNet Cardiovascular Signal Toolbox (Vest et al., 2018). A 60-second window with an increment of 60 seconds was implemented, and the default settings were used with the exception that the min/max inter-beat interval was set at 300/750 ms for the infant data and 300/1300 ms for the adult data. The Root Mean Square of Successive Differences (RMSSD) measure was taken to index Heart Rate Variability, but other frequency domain measures were additionally inspected and, as expected (Vest et al., 2018), showed highly similar results. To parse the actigraphy data we first manually inspected the data, then corrected artifacts specific to the recording device used, and then applied a Butterworth low-pass filter with a cut-off of 0.1 Hz to remove high-frequency noise.

## Justification for use of composite autonomic arousal measure

Previous research has identified strong patterns of tonic and phasic covariation between different autonomic measures collected from infants (Wass, Clackson, & de Barbaro, 2016; Wass, de Barbaro, & Clackson, 2015). Here, we include plots showing that the present analyses replicated and extended these results. The plots only show the sections of the data when participants were at home, comparing sections in which the infants were awake and asleep. Figure S1a shows cross-correlation plots examining the relationship between heart rate and movement. In both waking and sleeping sections the zero-lag correlation is 0.5. Figure S1c shows how these zero-lagged correlations vary on a per-participant basis. S1b shows an illustrative sample from a single participant. Sleeping sections show very low movement levels and lower heart rate. Of note, heart rate and movement do still inter-relate during the sleeping sections of the data (Figure S1c), albeit that the variability in heart rate and movement is lower. Figure S1 d-f show similar relationships between heart rate and heart rate variability, illustrating the strong and consistent negative relationships that were observed between these variables, as predicted. Based on these data, and following the approach we have taken in previous research (de Barbaro, Clackson, & Wass, 2016), we elected to calculate a composite measure of arousal for the analyses presented in the main text. This was done by calculating the natural logarithm of the actigraphy data, inversing the HRV data, epoching all three measures into 1Hz epochs, calculating a z-score separately for each participant and each measure, and then averaging the three measures into a single z-score.

Extensive previous research has identified fractionation, and differentiation, within our autonomic response systems (Lacey, 1967; Levenson, 2014; Lacey, 1967; Quas et al., 2014; Janig & Habler, 2000; Kreibig, 2010) – suggesting, for example, that the sympathetic and parasympathetic subdivisions may, to an extent, operate in a non-additive manner (Samuels & Szabadi, 2008). Although indubitably true, these findings should be seen as rendering incorrect our treatment of autonomic arousal as a one-dimensional construct here. Like many other arguments concerned with general versus specific factors, the question is rather one of the relative proportions of variance that can be accounted for by a single common factor in comparison with the variance accounted for by the sum of specific factors (Graham & Jackson, 1970; see Wass, 2018).


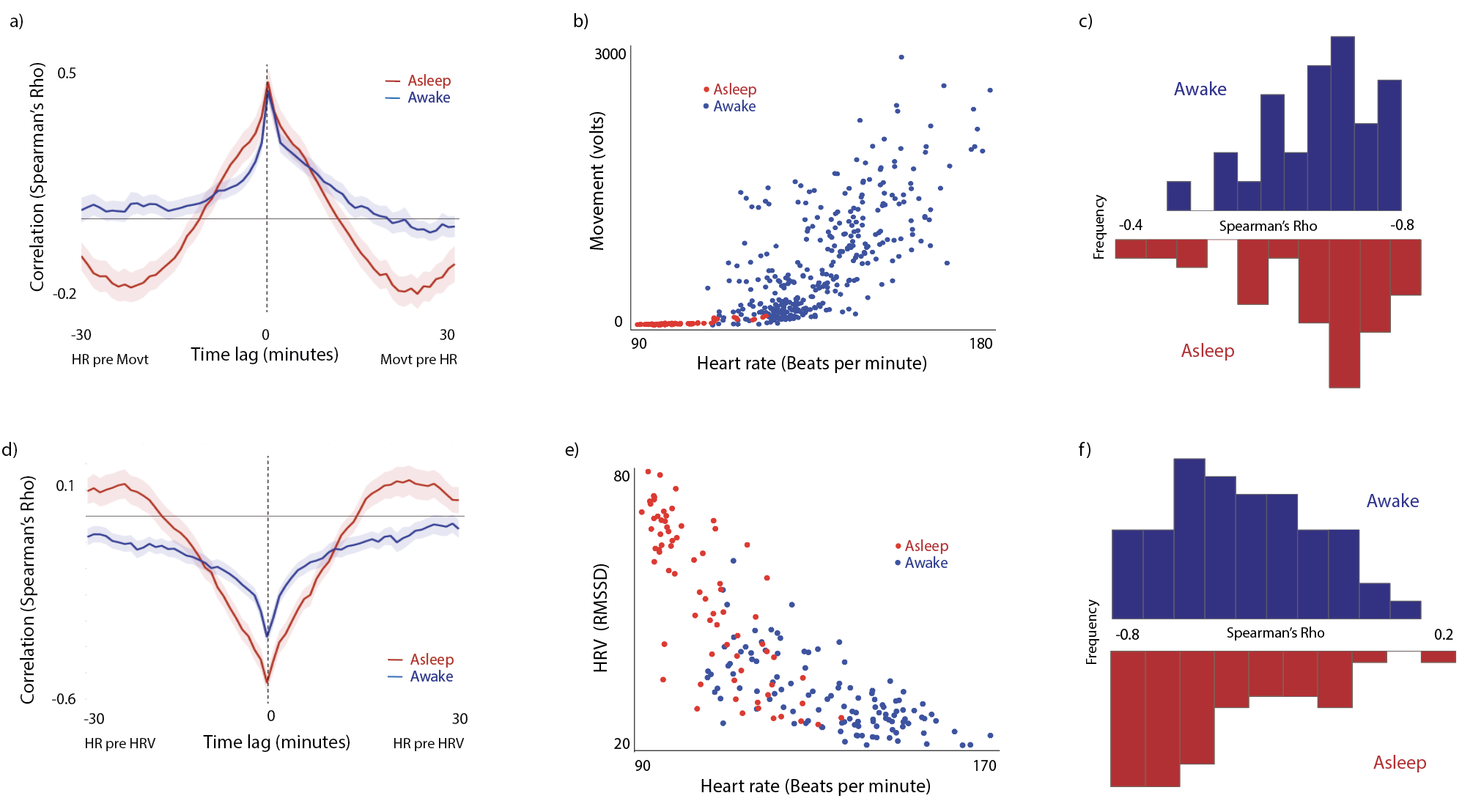


*Figure S1: Illustrating the relationship between the individual physiological measures included in the composite measure. a) Cross-correlation of the relationship between HR and Movement. b) Scatterplot from a sample participant. Each datapoint represents an individual 60-second epoch of data. c) Histograms showing the average zero-lagged correlation between 60-second epochs, calculated on a per-participant basis and then averaged. d)-f) Equivalent plots for Heart rate and Heart rate variability.*

## Home/Awake coding

Coding when participants were at home was performed using the GPS monitors built into the recording devices. The position of the participant’s home was calculated based on the postcode data that they supplied, and any GPS samples within a c.50m area of that location were treated as Home (corresponding to the accuracy of the GPS devices that we were using). To identify samples in which infants were sleeping, parents were asked to fill in a logbook identifying the times of infants’ naps during the day. This information was manually verified by visually examining the actigraphy and ECG data collected, on a participant by participant basis. Actigraphy, in particular, shows marked differences between sleeping and waking samples (see Supplementary Materials Figure S1 and So et al., 2005), which allowed us to verify the parental reports with a high degree of accuracy.

## Calculation of permutation-based temporal clustering analyses

To estimate the significance of the time-series relationships in the results, a permutation-based temporal clustering approach was used. Two different analytical approaches were used. One analysis (Method 1) looked at whether a ‘peak’ reactions were observed relative to a known “Time=0” moment (such as relative to a particular event). This was used for the analyses in Hypothesis 3.

The other analysis (Method 2) examined temporally contiguous patterns of change in instances where the centre-point of the expected response window is unknown, or unimportant (Maris & Oostenveld, 2007). This was used for the analysis in Hypothesis 1 and 4.

Method 1: This analysis examines whether the significant clusterings of elevated values around time=0 are observed. To estimate this, the following procedure was used. If y is Time=0, then for the first time interval (t=1) the observed data from y-1 to y+1 was excerpted (i.e., from 1 bin before to 1 bin after time=0). The proportional size of the excerpted data relative to the entire dataset was used to calculate a centile threshold (e.g. examining the central 10% of the data). The entire dataset was then rank ordered, and the highest 10% of the data was calculated. The proportion of highest data that was contained within the central segment of the data was calculated. The same calculation was then repeated for increasing values of t ranging from 1 to the total time window of the sample. Thus, for each value of t, if the observed data had been ‘perfectly’ ordered, with the highest value at time=0 and gradually decreasing values at increasing time lags, then the proportion of highest data contained within the central segment of data would always be 1. In this way, we quantified whether higher values were more commonly observed around the time=0 point in the data.

1000 random datasets were then generated with the same dimensions as the original input data. To ensure that the same level of autocorrelation was present in the simulated data as in the original datasets, multivariate autoregressive models were fitted to each sample included in the original dataset using the Matlab function ARfit.m (Neumaier & Schneider, 2001), and the matching AR parameters were used to generate each of the random datasets using the Matlab function ARsim.m (Neumaier & Schneider, 2001).

For each random dataset, the same series of calculations as described above was performed. In this way we estimated how, for each of the random datasets, the proportion of highest data contained within the central segment of the data varied across increasing time windows from the time=0 point. The results obtained from the random datasets were used to generate a histogram, and the likelihood of observed results have been obtained by chance was calculated by comparing the observed values with the randomly generated values using a standard bootstrapping procedure. Thus, a p value of <.01 indicates that the observed concentration of high values around the time 0 moment was observed in 10 or fewer of the 1000 simulated datasets generated for that time window.

Method 2: This analysis examines whether temporally contiguous patterns of change were observed in situations where the centre point of the expected response window is unknown or unimportant (Maris & Oostenveld, 2007). In each case, the test statistic (e.g. in the case of Figures 3a-c, an independent samples t-test) was calculated independently for each time window. Series of significant effects across contiguous time windows were identified using an alpha level of .05. 1000 random datasets were then generated using the same procedures as described for Method 1, above, the same sequence of analyses was repeated, and the longest series of significant effects across contiguous time windows was identified. The results obtained from the random datasets were used to generate a histogram, and the likelihood of observed results have been obtained by chance was calculated by comparing the observed values with the randomly generated values using a standard bootstrapping procedure. Thus, a p value of <.01 indicates that an equivalent pattern of temporally contiguous group differences was observed in 10 or fewer of the 1000 simulated datasets created.

# 2 Supplementary Results

## 2.1 Hypothesis 1 – Synchrony between infant and parent arousal – differences contingent on parental anxiety – further analyses

In addition to the analyses presented in the main text, we also examined how the association between parent and child arousal varies as a function of increasing the time-lag between the two variables (Fig 3b). In the low anxiety group, significantly negative associations were observed between parental arousal at time t and infant arousal at time t+6 to 10 minutes, suggesting that lower levels of parent arousal were associated with subsequently elevated levels of infant arousal (p<.001 after correcting for multiple comparisons using permutation-based cluster analysis (see SM section 1.2). This relationship was absent in the high anxiety group sample. No significant group differences were observed in the opposite direction, when considering how infant arousal forward-predicted subsequent levels of parental arousal. These results indicate that, in non-anxious parents, lower levels of parental arousal were associated with subsequently higher levels of infant arousal, and that this relationship was absent in the anxious parent group.


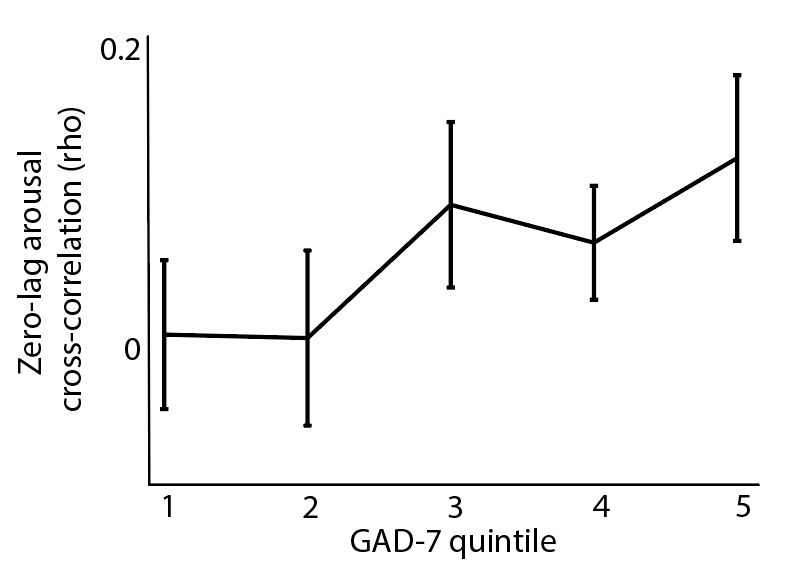


*Figure S2: Relationship of GAD-7 score to arousal cross-correlation (the same relationship shown in Figure 3a), but subdivided using a quintile split by GAD-7 score. The raw GAD-7 scores contained in each quintile group were: 1^st^ quintile – 0; 2^nd^ quintile – 1; 3^rd^ – 2; 4^th^ – 3 to 5; 5^th^ – 6-17.*

**References**

1. Blazer, D., Hughes, D., & George, L. K. (1987). Stressful life events and the onset of a generalized anxiety syndrome. *The American Journal of Psychiatry*, *144*(9), 1178–1183.
2. de Barbaro, K., Clackson, K., & Wass, S. (2016). Stress reactivity speeds basic encoding processes in infants. *Developmental Psychobiology*, *58*(5), 546-555.
3. Francis, J. L., Moitra, E., Dyck, I., & Keller, M. B. (2012). The Impact of Stressful Life Events on Relapse of Generalized Anxiety Disorder. *Depression and Anxiety*, *29*(5), 386–391.
4. Graham, F. K., & Jackson, J. C. (1970). Arousal systems and infant heart rate responses. In *Advances in child development and behavior,* (Vol. 5, pp. 59-117).
5. Jänig, W., & Häbler, H. J. (2000). Specificity in the organization of the autonomic nervous system: a basis for precise neural regulation of homeostatic and protective body functions. *Progress in brain research*, *122*, 351.
6. Kreibig, S. D. (2010). Autonomic nervous system activity in emotion: A review. *Biological psychology*, *84*(3), 394-421.
7. Lacey, J. I. (1967). Somatic response patterning and stress: Some revisions of activation theory. *Psychological stress: Issues in research*, 14-37.
8. Levenson, R. W. (2014). The autonomic nervous system and emotion. *Emotion Review*, *6*(2), 100-112.
9. Maris, E., & Oostenveld, R. (2007). Nonparametric statistical testing of EEG-and MEG-data. *Journal of neuroscience methods*, *164*(1), 177-190.
10. Muhsen, K., Lipsitz, J., Garty-Sandalon, N., Gross, R., & Green, M. S. (2008). Correlates of generalized anxiety disorder: Independent of co-morbidity with depression. *Social Psychiatry and Psychiatric Epidemiology*, *43*(11), 898-904.
11. Neumaier, A., & Schneider, T. (2001). Estimation of parameters and eigenmodes of multivariate autoregressive models. *ACM Transactions on Mathematical Software (TOMS)*, *27*(1), 27-57.
12. Quas, J. A., Yim, I. S., Oberlander, T. F., Nordstokke, D., Essex, M. J., Armstrong, J. M., ... & Boyce, W. T. (2014). The symphonic structure of childhood stress reactivity: Patterns of sympathetic, parasympathetic, and adrenocortical responses to psychological challenge. *Development and psychopathology*, *26*(4 0 1), 963-982.
13. So, K., Buckley, P. A. T., Adamson, T. M., & Horne, R. S. (2005). Actigraphy correctly predicts sleep behavior in infants who are younger than six months, when compared with polysomnography. *Pediatric research*, *58*(4), 761-765.
14. Vest, A. N., Da Poian, G., Li, Q., Liu, C., Nemati, S., Shah, A. J., & Clifford, G. D. (2018). An open source benchmarked toolbox for cardiovascular waveform and interval analysis. *Physiological measurement*, *39*(10), 1-23.
15. Wass, S. V., Clackson, K., & de Barbaro, K. (2016). Temporal dynamics of arousal and attention in 12‐month‐old infants. *Developmental psychobiology*, *58*(5), 623-639.
16. Wass, S. V., de Barbaro, K., & Clackson, K. (2015). Tonic and phasic co-variation of peripheral arousal indices in infants. *Biological Psychology*, *111*, 26-39.
17. Wass, S. V. (2018). How orchids concentrate? The relationship between physiological stress reactivity and cognitive performance during infancy and early childhood. *Neuroscience & Biobehavioral Reviews*, *90*, 34-49.
